# Plant-Based Green Synthesis of Nanoparticles: Production, Characterization and Applications

**DOI:** 10.3390/biom12010031

**Published:** 2021-12-25

**Authors:** Christophe Hano, Bilal Haider Abbasi

**Affiliations:** 1 Laboratoire de Biologie des Ligneux et des Grandes Cultures (LBLGC), INRAE USC1328, Eure et Loir Campus, Université d’Orléans, 28000 Chartres, France; 2 Department of Biotechnology, Quaid-i-Azam University, Islamabad 45320, Pakistan

## 1. Introduction

Nanotechnology is a fast-expanding and multidisciplinary field with many applications in science and technology [1,2,3,4,5,6,7,8,9,10,11,12,13]. This field combines key concepts from a variety of disciplines, including chemistry, engineering, physics, and biology, in order to provide novel methods for controlling and generating nanoparticles (NPs). These NPs are particles with at least one dimension ranging from 1–100 nm. Nanotechnology deals with the synthesis, characterization, and applications of a variety of NPs. Noble metals, such as gold, silver, or platinum, are commonly used to synthesize NPs by a variety of chemical and physical techniques; however, these processes are not ecologically friendly [1,4,9,14,15,16]. There is a pressing need to develop a non-toxic, environmentally friendly NPs production technology. Several safe, easy, cost-effective, reproducible, and scalable green synthesis approaches for NPs have been developed in recent years, inspired by the safety-by-design concept. As a result, several biological systems, such as yeast, fungus, bacteria, and plant extracts, are currently extensively employed in green synthesis approaches for the generation of NPs [1,9]. Plant-based NP green synthesis is now regarded as a gold standard among these green biological techniques owing to its ease of use and the diversity of plants. This work serves both as editorial for the present Special Issue, composed of two reviews and sixteen research articles, as well as a brief overview of current trends in green synthesis, characterization, and applications of a range of plant-derived NPs.

## 2. Green Synthesis and Characterization of Plant-Derived NPs

During the last decade, the concept of “Green Chemistry” for “Sustainable Development” has been widely investigated [17]. Sustainable development is described as development that meets the current demands while also balancing the ability of future generations to satisfy their needs [18]. Due to its concern with the evidence of pollution and the indiscriminate use of natural resources, sustainable development is especially important for various chemistry-based sectors [19]. The selection of a green or environmentally friendly solvent (the most widely used are water, ethanol, and their mixtures), a suitable non-toxic reducing agent, and a safe substance for stabilization are the three most important requirements for the green synthesis of NPs. Indeed, extensive synthetic pathways have been used to produce nanoparticles, with physical, chemical, and biosynthetic routes being the most popular. Chemical procedures are generally excessively costly and involve the use of toxic and hazardous chemicals that entail a variety of environmental risks [20]. In contrast, green synthesis is a safe, biocompatible, and environmentally friendly green method of synthesizing NPs for various applications, including biomedical uses [21]. Fungi, algae, bacteria, and plants have been used to carry out this green synthesis. However, plant components, including leaves, fruits, roots, stems, and seeds, have been widely utilized to synthesize different NPs [22]. Indeed, plant extracts have the ability to produce NPs with defined size, shape, and composition. Furthermore, the presence of a wide array of phytochemicals in their extract may function as natural stabilizing and/or reducing agents for NPs production. It is accepted that plant-derived NPs are also less likely to cause harmful side effects in humans when compared to chemically synthesized NPs, and exhibit a high biological potential with applications in agriculture, food science and technology, bioengineering, cosmetic or nanomedicine, and human health protection.

It is essential that these NPs be precisely and thoroughly characterized in order to ensure reproducibility in their production, biological activity, and safety. For this purpose, a wide range of physicochemical methods are used to very precisely characterized the synthesized NPs including ultraviolet-visible spectroscopy, Fourier transform infrared spectroscopy (FTIR), attenuated total reflection (ATR), Raman spectroscopy, photoluminescence analysis (PL), dynamic light scattering (DLS), UV-visible diffuse reflectance spectroscopy (UV-DRS), transmission electron microscopy (TEM), scanning electron microscopy (SEM), atomic force microscopy (AFM), field emission scanning electron microscopy (FE-SEM), X-ray diffractometer (XRD), X-ray photoelectron microscopy (XPS), energy dispersion analysis of X-ray (EDAX), thermal gravimetric differential thermal analysis (TG-DTA), or nuclear magnetic resonance (NMR) [23,24,25,26,27,28,29,30,31,32,33,34,35,36,37,38].

## 3. An Overview of the Different Types of Plant-Derived NPs

Different types of plant-derived NPs are presented, and their synthesis, characterization, and applications are discussed and published in this Special Issue.

Plant-based silver nanoparticles (AgNPs) are among the easiest to prepare [24,26,27,30,35,38]. For the green synthesis of silver nanoparticles, a silver metal ion solution and a reducing biological agent are required. The easiest and least expensive method for producing AgNPs is to reduce and stabilize Ag ions using a mixture of biomolecules, such as polysaccharides, vitamins, amino acids, proteins, phenolics, saponins, alkaloids, and/or terpenes [39]. Almost all plants have the potential to be exploited to prepare AgNPs.

Gold nanoparticles (AuNPs) have received tremendous attention because of their facile synthesis, easy surface functionalization [40], and unique characteristics, such as their high potential for use in medicine [41], low toxicity [42], and highly biocompatible nature [43]. Various chemical moieties in biogenic complexes operate as reducing agents in the production of gold nanoparticles, resulting in the reduction of gold metal ions and the formation of nanoparticles. Some studies have revealed that biomolecules, such as flavonoids, phenols, protein, and others, have an important role in the reduction of metal ions and the topping of gold nanoparticles in plant extracts [24,37].

Zinc oxide nanoparticles (ZnONPs) have received considerable attention over the last years because of their wide array of potential applications in biomedicine, cosmetic, optics, and electronics. Thus far, several investigations on the synthesis and utilization of ZnONPs by plants, microorganisms, and other species have been reported. Many studies have raised interest in their low-cost, safe, and simple synthesis. ZnONPs may be made from a variety of plant components, including flowers, roots, seeds, and leaves. Remarkably, these nanoparticles exhibit a high exciton binding energy of 60 meV and a huge bandgap of 3.37 eV, giving them a wide range of semiconducting properties [28,31,32,36].

Copper (Cu) is a comparatively low-cost metal that is more cost-effective than Au and Ag, and CuNPs have been synthesized by the reduction of aqueous Cu ions by different plant extracts [8,33]. The existence of a 578-nm peak on a UV-visible spectrometer, in particular, confirms their formation [44]. However, numerous questions about their biosafety persist [8].

Other metals, such as nickel (Ni) [29] or manganese (Mn) [25], are also presented. Note that some additional metals, such as titanium (Ti), palladium (Pd), cerium (Ce), or platinum (Pt), have lately been employed to prepare plant-based NPs with various biomedical or industrial applications [1,9,45].

## 4. Applications of Plant-Derived NPs

### 4.1. An Overview of the Potential Applications of Plant-Derived NPs

NPs are currently in high demand commercially due to their wide range of applications in industries, electronics, environment, energy, and more particularly in biomedical fields. NPs, such as the most commonly known Ag and Au NPs, have been widely explored in this sector and are of tremendous interest for biological applications. In general, plant-derived green NPs are also less likely to cause severe side effects in humans when compared to chemically synthesized NPs, and have a great application potential with applications in a variety of areas, including but not limited to:

- Nanomedicine and human health protection (antimicrobial, antiparasitic, antiproliferative, pro-apoptotic, pro- or anti-oxidative depending on the context, anti-inflammatory activities, etc.) [2,4,10,13,45];
- Agriculture (precision farming with controlled release of agrochemicals, target-specific delivery of biomolecules, more efficient nutrients absorption, detection and control of plant diseases, etc.) [3,46];
- Food science and technology (processing, storage, and packaging processes), in bioengineering (biocatalysts, photocatalysts, biosensors, etc.) [5];
- Cosmetics (sunscreen, anti-aging, hair growth, bioactive compounds delivery, nano-emulsion, etc.) [7].

With two review papers dealing with algae-based NPs synthesis and CuNPs, the current Special Issue sheds light on two less investigated tools and methodologies of green plant-based nanotechnology [6].

Algae are definitely ideal candidates for the green synthesis of NPs because they are rich in secondary metabolites that act as reducing and capping agents. Many potential applications have been already described including antimicrobial or anticancer actions, but also as antifouling, bioremediation or biosensing agents. However, unlike terrestrial medicinal and aromatic plants, algae were underutilized in the beginning of studies on the green synthesis of NPs using plant extracts. As this sector is still in its onset, scaling up for commercial applications is still challenging [6].

Cu is a relatively low-cost metal that is for example more cost-effective than Au and Ag. CuNPs have been produced via the reduction of aqueous Cu ions by various plant extracts. The review by Letchumanan et al. [8] provides a very comprehensive overview and current update of plant-mediated Cu/CuO (Cu oxide) NPs, covering their synthesis, therapeutic uses, and mechanisms. Although Cu/CuO NPs have a variety of therapeutic benefits, their toxicity to normal cells and important organs in humans might have significant adverse effects. As a result, prior to the use of these NPs in medicine, this potential toxic issue should be extensively examined. The toxicity of these NPs, as well as their effectiveness in comparison to commercial NPs in both in vitro and in vivo research, are reviewed and discussed [8]. This review also sheds light on the future prospects for producing plant-based Cu/CuO NPs as a therapeutic agent for a variety of diseases (including microbial infection, cancer, wounding, or inflammation) [8].

### 4.2. Anti-Cancer Potential

Nanomedicine is the use of nanotechnology in the treatment, screening, and diagnosis of a variety of diseases, including cancer [10,11,12,47]. It adds complete procedures and effective approaches against cancer through cancer prediction and diagnostics, prevention and medication, as well as possible individualized therapy [10,12].

Many plant-derived NPs have shown some potential against cancer cells. ZnONPs produced from a *Cassia auriculata* leaf extract, in particular, has shown tumoricidal activity against MCF-7 breast cancer cells while having no detrimental effect on normal MCF-12A human breast cells [34].

Similarly, green AuNPs produced from a *Trachyspermum ammi* seed extract inhibited cellular growth in HepG2 cancer cell lines in a concentration-dependent manner, which was linked to a reactive oxygen species (ROS)-driven apoptosis [37]. This mechanism has recently been reported to be potentially connected to mitochondrial action via ROS-induced Caspase-3 gene expression and enzyme activity following mitochondrial membrane potential disruption caused by plant-based NPs [11,47].

However, in addition to a deeper understanding of the molecular mechanism of action of NPs against cancer cells, there is also a need to properly understand the fate of NPs. These questions include how long NPs stay in the body, what conditions influence the duration of NP degradation, how to make NPs stay for longer or shorter periods, what are the long-term and short-term effects of NPs, how the body behaves towards these outsider entities on a micro and macro level, and how we can standardize NPs to ensure experiment reproducibility. These should be solved before introducing nanotechnologies into the healthcare industry. Aside from this, there are several questions that require further research and testing. In order to avoid any unanticipated consequences, we must also determine the possible risks linked with these nanomaterials. Furthermore, in order to obtain the safest and most successful therapy regimen, the numerous nanomedicines and nanoformulations targeting specific cancer cells must be thoroughly constructed. We conclude with the hope that nanotechnology will propel the development of more viable medicines to treat cancer, as well as offer researchers with powerful tools to overcome several bottlenecks in this health sector.

### 4.3. Anti-Leishmanial Potential

Leishmaniasis is a protozoan vector-borne illness that affects almost 350 million people worldwide. Chemotherapeutic medicines were initially used to treat leishmaniasis, but they had adverse side effects. Due to their unique properties, such as bioavailability, reduced toxicity, targeted drug delivery, and biodegradability, a variety of nanotechnology-based techniques and products have emerged as anti-leishmanial drugs, including liposomes, lipid nano-capsules, metal and metallic oxide nanoparticles, polymeric nanoparticles, nanotubes, and nanovaccines [2]. AgNPs containing xylan (also known as nanoxylan) synthesized in a green synthesis route with corncob xylan as a reducing and stabilizing agent demonstrated effective inhibitory activity against *Leishmania amazonensis* promastigote viability, whereas xylan alone had no effect [35]. This work nicely illustrates the potential of the nanoxylan as a promising new type of antiparasitic agent [35].

### 4.4. Antimicrobial Potential

Antibiotic resistance is one of the most pressing issues of recent years, and it is only going to become worse. Bacteria have developed resistance to antimicrobial agents as a result of the rapid evolution of the bacterial genome. Thus, in the search for a new therapy, biogenic NPs have shown encouraging results in the treatment of multidrug-resistant bacteria and might be a potential choice in the fight against such resistant pathogenesis [1]. To improve the antimicrobial response, NPs and other conjugates have been combined with different organic and inorganic compounds.

Ag has long been known for its antibacterial properties against a variety of bacterial strains. In particular, green AgNPs prepared from a *Carissa carandas* leaf extract demonstrated antibacterial efficacy against a variety of human pathogenic bacteria, with Gram-negative bacteria, particularly *Shigella flexineri* responsible for shigellosis, being more likely to be inhibited [27]. Similarly, bimetallic nanostructures coated with reduced graphene oxide generated from a stevia leaf extract, such as Pd-Ag nanostructures, can limit the development of Gram-negative bacteria *Escherichia coli* [23]. AgNPs obtained from the Saudi Arabian desert plant *Sisymbrium irio* showed potent inhibition potential against multidrug-resistant *Pseudomonas aeruginosa* and *Acinetobacter baumanii* that are responsible for ventilator-associated pneumonia [38]. Furthermore, antifungal activity of nanoxylan derived from corncob xylan against *Candida albicans*, *Candida parapsilosis*, and *Cryptococcus neoformans* has been described [35], whereas AgNPs obtained from the leaf extract of *Clerodendrum inerme* showed a dual antibacterial and antifungal actions against a wide range of human pathogenic strains [24].

Interestingly, AuNPs produced from the same *C. inerme* extract also showed very similar inhibition capacity [24]. The authors concluded that these NPs may have improved antimicrobial activity due to the synergistic effect of biologically active absorbed phytochemicals from this plant [24]. Antibiofilm action of AuNPs produced from a *T. amni* seed extract was also observed against *Listeria monocytogenes* and *Serratia marcescens*, most likely as a result of intracellular ROS production [37].

ZnONPs also showed potential antimicrobial activity as evidenced by the action of ZnONPs derived from a *Cinnamomum verum* bark extract against *E. coli* and *Staphylococcus aureus* [13]. Similarly, ZnONPs derived from a *C. auriculata* leaf extract exhibited antibacterial activity due to direct cell contact, which disrupted bacterial cell integrity [34].

Other metallic NPs, such as CuONPs derived from *Cymbopogon citratus*, can exhibit significant antimicrobial activity, including antibiofilm properties [33]. Interestingly, these authors noted a variation in antibiofilm activity, which they suspect is due to differences in the cell wall compositions of the examined bacterial strains [33]. MnONPs derived from an *Abutilon indicum* leaf extract demonstrated potent antibacterial activity against both Gram-negative and Gram-positive bacteria [25], whereas NiONPs deriving from stevia leaf extract were more effective against Gram-negative bacteria [29]. This shows that antimicrobial activity is influenced by the type of NPs produced, but also the composition of the coated phytochemicals on their surfaces, which is affected by the plant extract used for NPs synthesis.

Cell wall disruption, cell membrane disintegration, massive free radical production, specific (targeted) and/or specific actions against proteins, DNA fragmentation, vital enzyme inhibition, loss of cellular fluids, and disruption in electron transport have all been proposed as possible mechanisms for NPs antibacterial activity [1]. Bio-mediated NPs might also have an antifungal effect by causing excessive ROS generation. However, few studies have focused only on fungus as of yet [1]. Despite advances in understanding of the antimicrobial efficacy of plant-based NPs, much remains unclear regarding their specific mechanism of action, toxicity, and possible environmental issues.

### 4.5. Agricultural Applications

When agricultural pathogens are targeted, the antimicrobial activity outlined in the previous Section may be effective for crop protection. In particular, ZnONPs have demonstrated their wide agricultural interest showing an anti-phytopathogenic action against both bacteria as evidenced by ZnONPs derived from lemon fruit against soft rot bacteria pathogen *Dickeya dadantii* [31], and fungi as illustrated by the fungicidal activity of ZnONPs produced using a *Eucalyptus globules* extract against major pathogens of apple orchards [32]. It is noteworthy that TiO_2_NPs produced from lemon fruit showed antibacterial activity comparable to ZnONPs against *D. dadantii* [31].

Through modifying abscisic acid concentration, ion homeostasis, and defense mechanisms comprising both enzymatic and non-enzymatic antioxidants, AgNPs synthesized from a wheat extract significantly contributed to alleviate the negative effects of salinity stress in wheat [30]. Interestingly, ZnONPs exhibited low toxicity and the capacity to stimulate the antioxidant response of flax seedlings as well [28].

### 4.6. Antioxidant Action

Excessive oxidative stress generated by the action of mitochondria and other internal or external sources may result in oxidative damages to various cell macromolecules (membrane lipids, proteins, and DNA), leading to functional declines, degenerative diseases, and aging [48]. Antioxidants may be able to reverse this detrimental process and may be used to treat aging and age-related diseases. Some green plant-derived NPs have been described for their antioxidant potential as shown for AgNPs produced from a *C. carandas* leaf extract [27], AuNPs and AgNPs deriving from a *C. inerme* leaf extract [24], or NiONPs prepared from a stevia leaf extract [29]. The phytochemicals coated on the NPs surface have certainly a prominent influence in the observed antioxidant action. Commonly, just one in vitro assay, such as the DPPH (2,2-diphenyl-1-picrylhydrazyl) assay, is performed. However, due to the complex nature of phytochemicals, and in particular, because the determination of antioxidant activity is significantly reliant on the reaction mechanism involved, antioxidant activity of should not be measured using a single approach [49]. Therefore, the validity of the results from in vitro cell-free antioxidant tests must be restricted to the interpretation in terms of chemical reactivity, but in vivo (cellular) validation is strongly required.

### 4.7. Other Applications

Other potential applications, such as (photo)catalytic and/or absorption potential applications, are also described. AgNPs produced by *Matricaria chamomilla* showed effective catalytic activity against Rhodamine B under UV light, which could make it a promising material for wastewater treatment [26]. MnONPs produced from an *Abutilon indicum* leaf extract has shown efficient absorption activity against the heavy metal CrVI as well as strong photocatalytic activity, indicating the potential to remediate various organic and inorganic contaminants [25]. Finally, the photocatalytic H_2_ production, mediated by Pd-Ag bimetallic nanostructures coated with reduced graphene oxide produced from a stevia leaf extract, can be noted [23].

## 5. Conclusions and Future Directions

The growing demand for green chemistry and nanotechnology has pushed for the development of green synthetic methods for the production of nanomaterials using plants, microbes, and other natural resources. Researchers have been focusing on the green synthesis of NPs, using an environmentally favorable technique. Due to their cost-effectiveness, nontoxic approach, simple availability, and ecofriendly nature, considerable research has been conducted on plant extract-mediated NPs production and their prospective uses in numerous industries. Plants have a variety of unique compounds that help in the synthesis process and accelerate the synthesis kinetic. The use of plants for green nanoparticle synthesis is an interesting and emerging aspect of nanotechnology that has a significant impact on the environment and contributes to nanoscience’s long-term sustainability and progress. Catalysis, medicine, cosmetic, agriculture, food packaging, water treatment, dye degradation, textile engineering, bioengineering sciences, sensors, imaging, biotechnology, electronics, optics, and other biological sectors are just some of the potential applications of these green plant-based NPs. These NPs might be the future impetus for the biomedical field in the drug delivery system. These green NPs might be also employed in a variety of ways, including phytopathogen treatment in agriculture or water disinfection for environmental cleanup. This green approach of NPs synthesis is becoming more popular and is expected to develop exponentially in the future; nevertheless, long-term impacts on animals and humans, as well as the accumulation of these NPs in the environment and their influence, must be addressed in the future. This Special Issue gathered cutting-edge research and review articles on the plant-based green synthesis of NPs, their production, characterization, and applications, with the goal of providing the most comprehensive overview of all these features and future challenges.

## Data Availability

All the data are included in the present study.
